# Notes and News

**Published:** 1897-08-28

**Authors:** 


### NOTES AND NEWS.

(Collected and Communicated.)

The International Medical Congress at Moscow and the meeting of the British Medical Association at Toronto are now being held.

Mr. H. E. W. Hoffmeister, M.R.C.S., L.R.C.P., has been appointed Surgeon-Apothecary to the Queen at Osborne, in conjunction with Mr. W. Hoffmeister, M.D., and Mr. J. B. Hoffmeister, M.R.C.S., L.R C.P.

The Queen is to be invited to lay the foundation-stone of the new infirmary at Newcastle in October on her return from Balmoral. If Her Majesty is unable to comply, some member of the Royal Family will be invited to act for her.

Water drinkers in London will not be consoled when they learn that the Welsh Harp Reservoir is in a far from perfect condition. Twice during the last two years the destruction of large quantities of fish is reported, owing to the polluted condition of the water.

The annual general meeting of the British Dental Association has been held at Dublin. Some interesting demonstrations took place, and a large number of papers were read. Dr. Theodore Stack acted as president, and a variety of entertainments were held in honour of the association.

The Uruguay Legislature have presented £2,000 and the honour of citizenship to Dr. Sanarelli in recognition of his discovery of the microbe of yellow fever. Expressions of regret that the recognition cannot be larger owing to the poverty of the country accompany the awards. Dr. Sanarelli has open before him possibilities of greater things elsewhere, as the Legislature of Brazil offers an award of £22,000 for a work demonstrating the existence of the bacillus, and a like sum for a practical work on the treatment of the disease. The discoverer of a curative serum will be offered the post of director of an establishment, which the Government of Brazil is prepared to support, for the preparation of such serum.

Two new wards have been opened at the Blackpool Hospital by the Home Secretary.

Earl Percy recently opened the new wing at the Tynemouth Victoria Jubilee Infirmary, North Shields. This wing is the gift of Colonel R. S. Donkin, M.P.

The vestry of St James's, Westminster, have contributed the sum of £150 to the Prince of Wales's Fund out of the surplus profit derived from the grand stand erected in Waterloo Place to view the Jubilee Procession.

The foundation-stone of a cottage hospital at Emsworth has been laid. The amount necessary for the building of the institution has been collected, but an endowment is still wanting. The hospital is to contain two wards with three beds in each, a day room, emergency ward, and the usual offices.

Lord Tredegar recently laid the foundation-stone of a new county hospital at Newport, in Wales. Towards this institution Dr. Garrod Thomas, consulting physician of the old hospital, gave £5,000, and Lord Tredegar the site. At the ceremony many contributions were announced.

A gentleman, who prefers to remain anonymous for the present, has offered to build at his own cost the proposed Victorian Convalescent Home for Women—Surrey's memorial of the Queen's reign—provided £800 per annum is secured through endowment and annual subscriptions.

The Local Government Board have issued a report on the preparation and storage of glycerinated calf vaccine lymph. Sir R. Thorne-Thorne, who has written the introduction to the report, recommends that vaccination carried out under the Government should be performed exclusively with calf lymph, and that a system of calf-to-arm vaccination should be instituted and applicable when desirable, and to afford facilities of comparison with those vaccinations performed with preserved lymph. He further recommends that the distribution of calf vaccine through Government sources should be limited to glycerinated or similar preparations of lymph and pulp material in air-tight tubes or glass receptacles. Finally, he recommends the reorganisation of the Local Government Board's vaccine station, constructively and administratively, and that the laboratory should be placed under the direct supervision of a bacteriological expert.

The twelfth International Medical Congress opened at Moscow on August 19th in the Imperial Theatre, in the presence of the Grand Duke Sergius, patron of the Congress and the president of the Committee of Organisation. Professor Sklifossowsky delivered the opening address. Professor Virchow and Mr. Lauder Brunton both read papers. A fête was held in the Galleries of Commerce, and during the subsequent days many entertainments and excursions were arranged. A full programme of proceedings, together with all necessary information, was published every day in the official journal of the Congress. Special excursions to places of interest in the city were organised by the Ladies' Committee, presided over by the Grand Duchess Elizabeth Feodrowna. At the bureau in connection with this committee, ladies were in attendance daily to give necessary information and assistance to lady members of the Congress.

